# Smart Wireless Transducer Dedicated for Use in Aviation Laboratories

**DOI:** 10.3390/s24113585

**Published:** 2024-06-02

**Authors:** Tomasz Kabala, Jerzy Weremczuk

**Affiliations:** 1 Łukasiewicz Research Network–Institute of Aviation, 02-256 Warsaw, Poland; 2 Faculty of Electronics and Information Technology, The Institute of Electronic Systems, Warsaw University of Technology, 00-665 Warsaw, Poland; jerzy.weremczuk@pw.edu.pl

**Keywords:** smart sensors, smart transducers, measurements, aviation, wireless power transfer, wireless sensor network, telemetry, IoT (Internet of Things)

## Abstract

Reliable testing of aviation components depends on the quality and configuration flexibility of measurement systems. In a typical approach to test instrumentation, there are tens or hundreds of sensors on the test head and test facility, which are connected by wires to measurement cards in control cabinets. The preparation of wiring and the setup of measurement systems are laborious tasks requiring diligence. The use of smart wireless transducers allows for a new approach to test preparation by reducing the number of wires. Moreover, additional functionalities like data processing, alarm-level monitoring, compensation, or self-diagnosis could improve the functionality and accuracy of measurement systems. A combination of low power consumption, wireless communication, and wireless power transfer could speed up the test-rig instrumentation process and bring new test possibilities, e.g., long-term testing of moving or rotating components. This paper presents the design of a wireless smart transducer dedicated for use with sensors typical of aviation laboratories such as thermocouples, RTDs (Resistance Temperature Detectors), strain gauges, and voltage output integrated sensors. The following sections present various design requirements, proposed technical solutions, a study of battery and wireless power supply possibilities, assembly, and test results. All presented tests were carried out in the Components Test Laboratory located at the Łukasiewicz Research Network–Institute of Aviation.

## 1. Introduction

The quality of aviation testing is highly dependent on the functionality and reliability of measurement systems. Measured data are the basis for estimating critical limits, operability confirmation, or the further redesign of components. Two main types of tests are performed in aviation component test laboratories, namely performance tests, where engineers try to prove the limits of the tested components, and long-term endurance tests, in which wear and durability are investigated [1,2,3]. In both groups, the wiring of sensors and the setup of measurement systems are significant tasks for laboratory teams because potential mistakes could cause failures or incorrect conclusions to be drawn from test data. Therefore, it is essential to look for methods to optimize the test preparation process. Every tested component needs a different approach to test rig configuration. Moreover, there could be many test rig reconfigurations in one test campaign to fulfill the various test program requirements. Aviation laboratories are dominated by the classic approach to measurements, where current or voltage signals are sent to multi-channel measurement cards. The most popular sensor types used in aviation test instrumentation are thermocouples, RTDs, strain gauges, load cells, pressure sensors, flow meters, proximity sensors, and vibration sensors [4,5]. In some test campaigns where the test object is moving or rotating, engineers use telemetry systems [6,7] to transmit signals through radio waves or inductive coupling [8]. Still, the costs of these systems are high, and typically, they do not use standard wireless network protocols and other smart functionalities. The use of wireless smart transducers could give the functionality of smart sensors to the basic types of sensors. According to the IEEE 1451 standard [9], a smart transducer provides functions beyond those necessary to generate a correct representation of the measured or controlled quantity [10,11]. A smart transducer is a device that has one of the following functions:

- Network communication with other devices;
- Processing and analysis of measured data;
- Failure detection and self-diagnosis;
- Ability to make decisions or self-learning.

**Figure 1:**
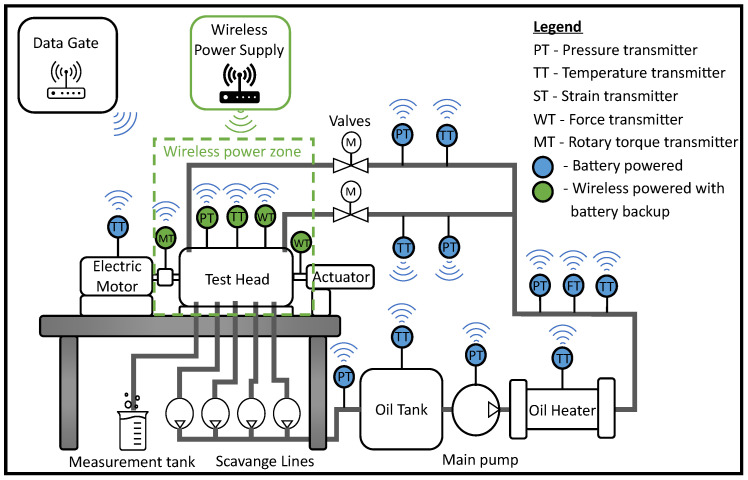
Concept of smart transducer network in rotating components laboratory.

Nowadays, smart wireless sensors are being widely introduced in most areas [12,13,14], including aircraft monitoring [15,16,17]. Despite this, their use in the area of aerospace component testing laboratories is very limited. The use of wireless smart transducers in aviation component test laboratories could change the means of test preparation, as illustrated in Figure 1.

The lack of wires between sensors and measurement cards improves the flexibility of the test rig configuration. Moreover, in a situation when one test is performed a moment after another test campaign, the instrumentation of the test head could be prepared and checked outside the test rig. When the test head is moved to the test room, transducers could be automatically connected to the data gate. In the same way, an instrumented test head or tested object could be installed in another laboratory and transmit the data without changing the accuracy. The classic approach to continuing testing in other laboratories brings about concerns about differing measurement accuracies. Shorter analog parts of measurement paths could also increase the accuracy of measurements and reduce the probability of mistakes connected with the incorrect configuration of measurement channels. Certification of measurements, which is one of the most important tasks for aviation laboratories [18], would be simplified because the transducer could be calibrated in combination with a sensor by an external laboratory. In the classic approach, some sensors must be calibrated separately from the measurement cards. The total error is estimated from the calibration certificate of the measurement card; the sensor; and, in some situations, a transducer between them [19]. For a pair consisting of a smart wireless transducer and a sensor, the laboratory could prepare one certificate with the digital output error of the tested system. Digital communication with a data gate has to be checked. Still, it would be enough to check the characteristic boundary points to prove wireless communication, and there should be no accuracy loss compared to the certificate. Most telemetry systems on the market are battery- or inductive-powered without a low-power design approach, which is connected with striving for high sampling frequencies [20,21]. However, not every test campaign needs high-speed measurements. Decreasing the power consumption of electronic devices and efficient power management make it possible to power smart sensors and other devices wirelessly from low-energy sources [22], and there are several ways to power devices wirelessly [23,24,25]. Still, the use of electromagnetic waves from the radio or microwave spectrum supported by a battery power supply seems to be most suitable for the powering of smart sensors in aviation laboratories because of the flexibility of sensor placement. Wirelessly transmitted power decreases significantly with the increasing distance between transmitter and receiver (with a power of two) [26]; however, it could be enough to power the transducers and sensors located in a small area. WPT (Wireless Power Transfer) based on electromagnetic radiation could extend the battery operation time or make it possible to supply the transducer without batteries over a distance many times further than typical inductive-powered systems [27,28]. On the other hand, low power consumption extends the battery operation of the smart transducer to months or even years, which could be enough to operate without wireless power transfer.

## 2. Materials and Methods

The following sections present the methods, technologies, and components used in the project of an innovative smart wireless transducer dedicated for use in aviation laboratories.

### 2.1. Basic System Concept

**Figure 2:**
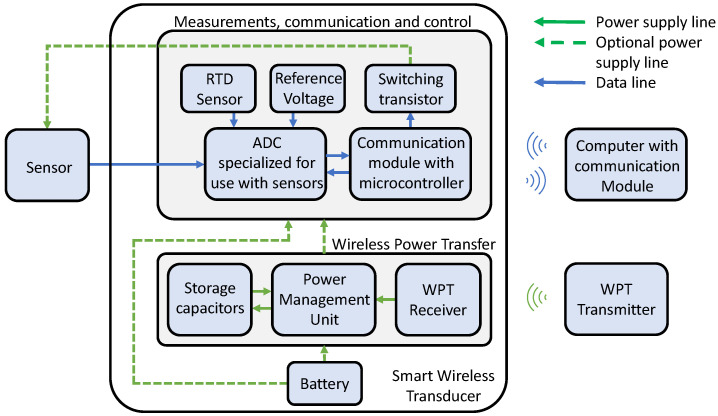
Basic concept of the designed system.

A review of wireless smart sensor technologies and an analysis of aviation laboratory requirements made it possible to prepare a basic diagram of the system structure, as shown in Figure 2. The main goal of the project was to design a smart wireless transducer that could operate with sensors typical for laboratory infrastructure and be used during testing of aircraft components. Generally the elements of the transducer can be divided into two groups. In the first group, we have two main components, namely the ADC (Analog-to-Digital Converter), which is used for operation with the sensors, and a communication module with an inbuilt microcontroller, which manages the operation of the system. The computer with the communication module receives the data wirelessly in real time, with the frequency defined in the settings of the transducer. A second group of elements is responsible for the power supply of the system. The transducer can be powered directly from the battery or wirelessly through a radio transmitter with a battery as a backup. In wireless power transfer mode, the PMU (Power Management Unit) manages the energy storage process and stabilizes the voltage.

### 2.2. System Structure

**Figure 3:**
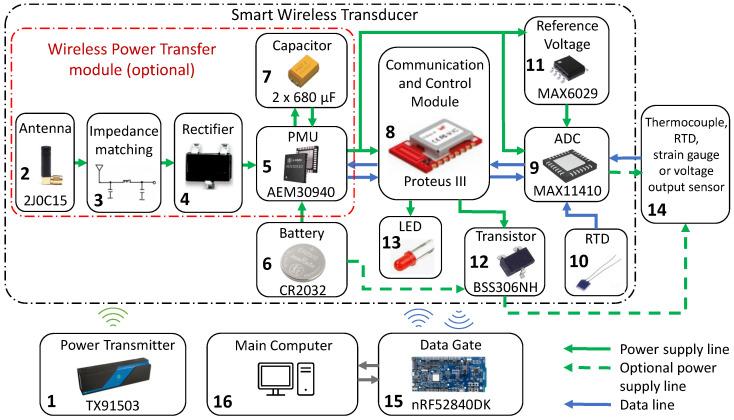
Structure of the designed telemetry system.

Based on the basic system structure presented in Section 2.1, components were selected, and the target system structure was developed, which is shown in Figure 3. The main parameters of the designed wireless smart transducer are summarized as follows:

- Communication through low-energy Bluetooth;
- Sampling in the range of 0.1–2 Hz (reduced due to power saving);
- Ability to work with thermocouples, RTDs, strain gauges, and voltage outputs;
- Combination of RF and battery power supply (to optimize the flexibility of use);
- Small size (17 × 35 mm)

When the system uses the WPT module, all circuits in the measurement system are powered by a 3 W, 915 MHz power transmitter (1), which interacts with a 3.3 dBi (maximum peak gain) replaceable antenna (2). The impedance matching network (3) increases the effectiveness of power transfer. The alternating voltage is transformed to DC form in the rectifier (4). Next, the PMU (5) manages the available sources of power supply. In a situation without access to a wireless power supply, the PMU switches to use the battery (6), which can be installed in the battery holder or outside the transducer. When the radio power source is available, the module uses only that source and stores excess energy in the capacitor (7), especially between measurement cycles when the system is in sleep mode. In both situations, the PMU increases and stabilizes the voltage used to power other circuits on the PCB, which enables the system to operate at a very low level of input voltage and extends the range of operation with the use of wireless power transfer. The system can also work without a WPT module with a jumper, which connects the battery directly to the power input of the main PCB. The main control communication module (8) is responsible for wireless communication, data processing, and control of the ADC (9). For temperature compensation, a reference temperature sensor (10) is used during thermocouple measurements. To increase the accuracy of measurements, the system uses a reference voltage source in the form of another circuit (11). A MOSFET transistor (12) switches the configurable power source during measurements with strain gauges and voltage output sensors. An LED (13) is used for debugging and basic communication with the user. All signals measured by sensors (14) are digitized and sent to the data gate (15), then to the main acquisition system of the laboratory (16) for further storage and processing.

### 2.3. PCB Design

**Figure 4:**
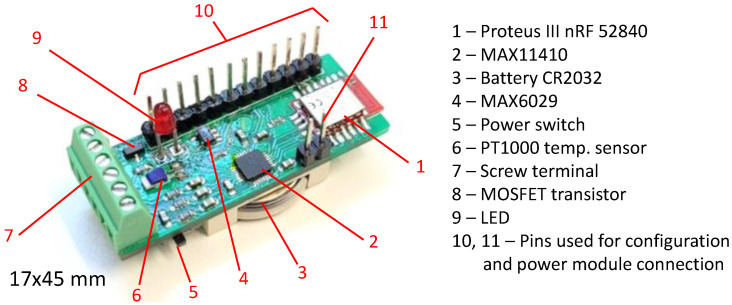
Measurements, data processing, and communication module.

**Figure 5:**
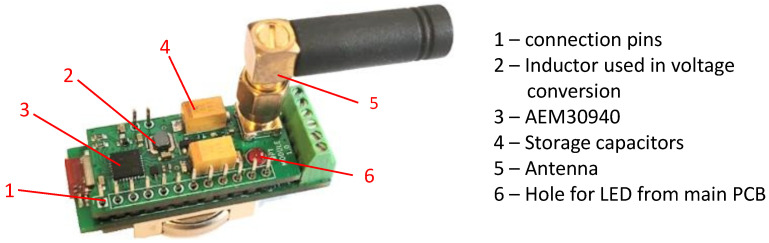
Wireless power transfer module installed on top of the main board.

To fulfill the project requirements presented in the previous sections, the transducer design was arranged in the form of two PCB boards. The first PCB board shown in Figure 4 plays the main role and is responsible for measurements, data processing, and communication. It can be used independently from the second board when the only source is a battery, but there is no voltage converter, so the battery can be used only in the range of 2.7–3.6V, which are the limits of electronic components. That range enables the base module to work with a lithium coin cell and various Li-Po and Li-Ion rechargeable batteries. The second optional board shown in Figure 5 is used for WPT and power management. The change in power supply source controlled by the transistor and reference voltage source is carried out by means of jumpers available to the user.

### 2.4. Microcontroller and Wireless Communication

**Figure 6:**
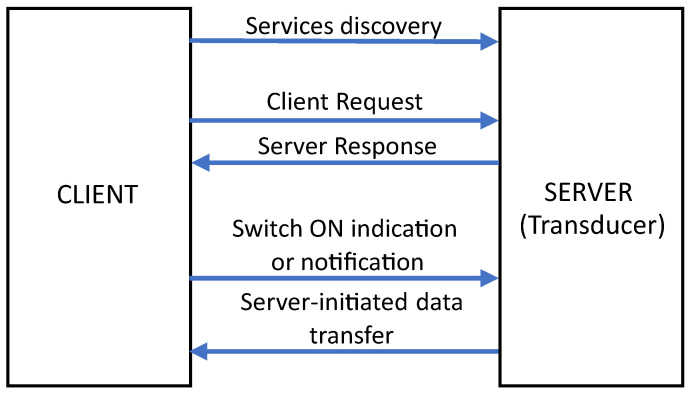
Data transfer through BLE.

The Proteus III module with the nRF52840 SoC (System on Chip) from Würth Elektronik (Germany), based on a 32-bit ARM Cortex M4 core [29,30], was used for communication, control of the ADC, and data processing. This small-size module (12 mm × 8 mm) can work with various wireless low-power communication standards, e.g., BLE 5.1, Thread 1.1, and Zigbee 3.0 [31,32,33]. The design work was focused on the use of BLE because of its high resistance to electromagnetic noise, low power consumption, and popularity. The goal of the project was to build a wireless smart transducer that can be part of a wide WSN (Wireless Sensor Network), but it should also be compatible with typical computers, tablets, and smartphones. In the simplest test campaigns, one of these devices could play the role of the central point of a network, where the data are stored and visualized. That approach simplifies test preparation because there is no need to build dedicated measurement cabinets connected by wires to the sensors. Basic point-to-point communication in BLE takes place between devices called peripheral and central devices [34]. To start communication, the peripheral device sends advertising packets to the central device, which periodically changes its state to scanning mode. The frequencies of advertising and scanning are configurable in the program settings. Higher frequencies increase the probability of connection, which causes a shorter time needed to start a connection event, but it also makes power consumption higher. Devices could also be additionally linked by the bounding process, introducing the possibility of automatic reconnection after the next startup. Keys are stored in the memory of the devices, and there is no need to perform the pairing process again, as it happens in the classic version of Bluetooth. After the connection process, the device takes on the roles of client and server, as shown in Figure 6.

Communication is carried out with frequency, which is set in the communication parameters. The client sends a request for data to the server, which can send the data in response. Data on the server are stored in the form of characteristics that represent data and services, which are sets of connected characteristics. Data from the server can be sent as a notification and indication (e.g., when the value was changed), but before that, the client has to send the request for these features. The program simulates serial communication with the use of two characteristics (RX and TX) for the transmitting and receiving of data. The implemented program is based on examples from the software development kit and BLE stack (soft device) for nRF52840, which are distributed by Nordic Semiconductor [35]. Communication with a higher number of devices could be implemented in a similar way. Devices could also play the roles of server and client at the same time during communication with different devices. These features make it possible to build a network in the structure of a star or tree [36]. There is also the possibility of building a mesh network with the use of another BLE stack, but it was not investigated in the described research. Literature research shows that BLE Mesh [37] offers a great opportunity to extend the range of BLE, but it is not a problem in the network of sensors placed in the same room. Moreover, the time of packet delivery could also be changed during the reconfiguration of the mesh network, which could be potentially problematic in correlation during test data analysis.

### 2.5. Measurements

**Figure 7:**
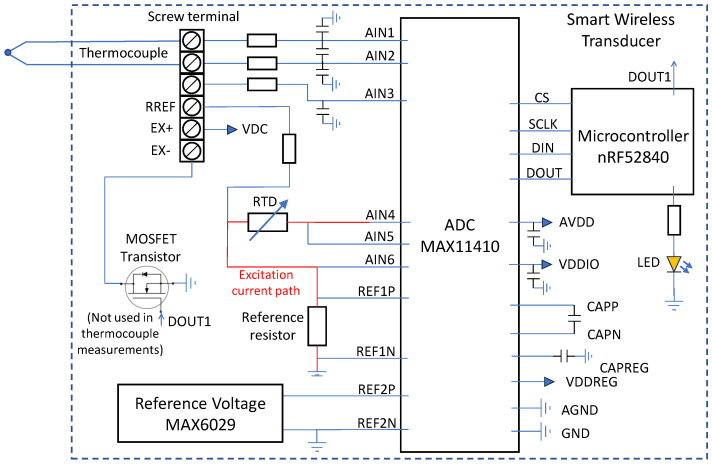
Application of MAX11410 for use with thermocouple.

One of main goals of the design was to enable the transducer to work with sensors typical for aviation test rig instrumentation. The design was focused on operation with thermocouples, RTDs, voltage output sensors, strain gauges, and sensors based on strain gauges, e.g., load cells or torque meters. For this purpose, the MAX11410 was selected, which is a 10-channel, 24 bit ADC specialized for use with sensors [38]. In comparison to general-purpose ADCs, it has features that facilitate the use of sensors, e.g., configurable current source for excitation, simplified temperature compensation, a configurable filter, and gain. The designed transducer uses six of the ten inputs of MAX11410, as shown in Figure 7. Three inputs are used to operate with an RTD installed on the PCB. The next three are connected to screw terminals for external sensor connections. Communication is performed through the SPI protocol operated by the implemented software controller. For each type of supported sensor, low power consumption is a result of low-power components and short operation time. The system wakes up from sleep mode only to perform measurements, perform calculations, and send data. In each case, the operation of MAX11410 consists of writing and reading registers through SPI according to the procedure prepared for a specific type of sensor and the proper jumper configuration used for the selection of power source and reference voltage.

To perform measurements with an RTD, one of the outputs generates current that flows through that sensor and a reference high-precision resistor. The voltage drop on a high-precision reference resistor plays the role of reference voltage for the ADC, so the measurement process is independent of the change in current. The value received through the SPI is the ratio between the actual resistance of the RTD and the resistance of the reference resistor multiplied by the maximum ADC bit range of 2^24^. To obtain a temperature value from the measured resistance of the PT1000 sensor bellows, Equation (1) was implemented [39].

(1) $$T_{RTD}=\frac{-R_{0}a+\sqrt{R_{0}^{2}a^{2}-4R_{0}b\left(R_{0}-R_{m}\right)}}{2R_{0}b}$$

where: $R_{0}$ = 1000, *a* = 3.9083 × 10^−3^, and *b* = −5.7750 × 10^−7^.

**Figure 8:**
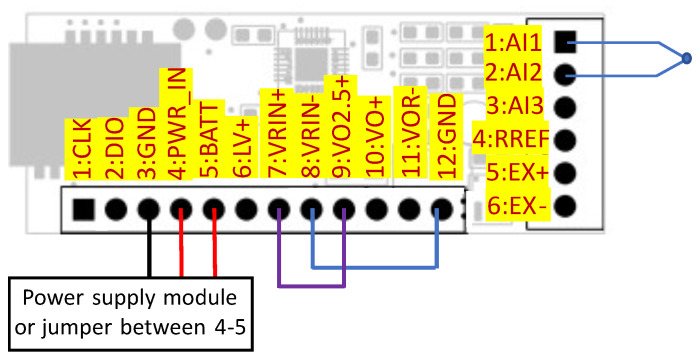
Operation with thermocouple.

The PT1000 sensor type is used for CJC (Cold Junction Compensation) of the thermocouple, which can be connected to screw terminal, as shown in Figure 8. To perform compensation, the measured temperature value of the junction is used to calculate the voltage of the hypothetical thermocouple using Equation (2) (thermocouple type K). Next, program calculates the sum of the voltage measured on the thermocouple wires (compared to the reference voltage from MAX6029) and the voltage calculated with the use of the RTD temperature [40]. The result is the argument of Equation (3) used to calculate the final temperature. Each type of sensor needs a specific configuration of pins, as listed in Table 1.

(2) $$E_{r}=\sum_{i=0}^{n}C_{i}{\left(T_{RTD}\right)}^{i}+\alpha_{0}e^{\alpha_{1}{\left(T_{RTD}-126.9686\right)}^{2}}$$

(3) $$T_{k}=c_{0}+c_{1}E_{k}+c_{2}E_{k}^{2}+\ldots c_{i}E_{k}^{i}$$

where: $T_{k}$ is measured temperature; $E_{r}$, $E_{k}$ are voltages; and $C_{i}$, $\alpha_{0}$, $\alpha_{1}$, and $c_{i}$ are coefficients.

**Figure 9:**
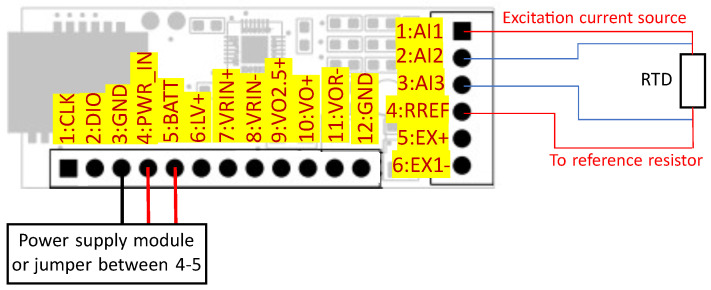
Operation with 4-wire RTD (AI1 used as an excitation current source).

The system can work not only with an inbuilt RTD sensor but also with external RTD sensors in a 2-, 3-, or 4-wire configuration, as shown in Figure 9. Additional wires are used to reduce the effect of wiring resistance, which could be significant for long measurement paths. In general, RTDs are more accurate than thermocouples, but thermocouples are more popular in aviation instrumentation due to their high measurement range, robustness, and various shapes.

**Figure 10:**
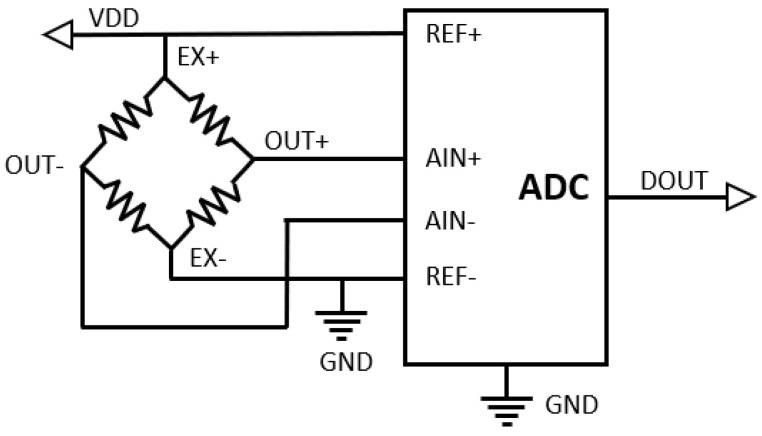
Excitation voltage used as a reference voltage for ADC (full bridge).

Measurements with strain gauges can be performed in a similar way as for RTD sensors, or the output voltage from the bridge can be measured. The equations used to calculate the strain for different strain gauge configurations (quarter, half, or full bridge) can be found easily in the literature [41,42] and are based on a combination of voltage measured across the strain gauge bridge, excitation voltage, the gauge factor for a specific type of strain gauge, and coefficients. For strain gauge-based sensors, it is also typical to specify the output in mv/V as a reference to power supply voltage. In bridge measurements, MAX6029, which is used for thermocouple measurements, can also play the role of reference voltage for ADC, but the measurements are dependent on changes in the excitation voltage level [43,44]. Therefore, to achieve higher accuracy, it is better to use the excitation voltage as a reference voltage. In the configuration shown in Figure 10, measurements are independent of power supply variations.

**Figure 11:**
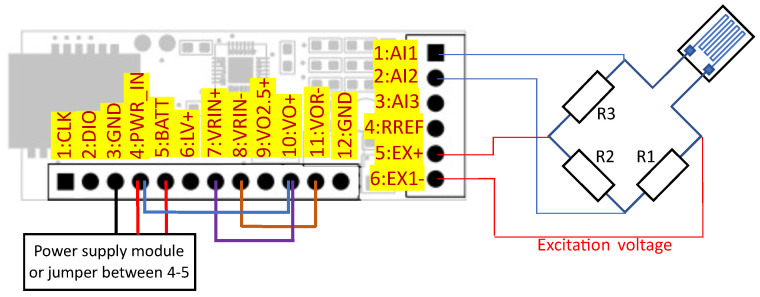
Operation with quarter-bridge.

**Figure 12:**
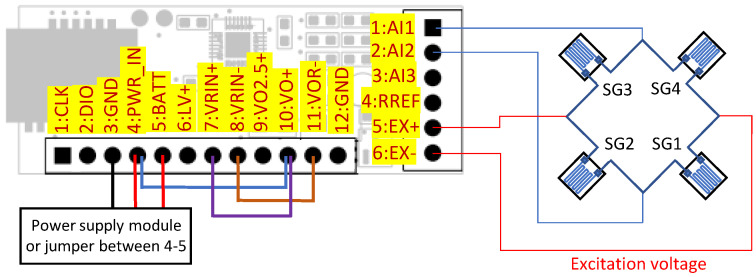
Operation with full-bridge.

The power consumption depends on the resistance, excitation voltage, and time of operation [45]. Constant use of strain gauges generates excess power consumption for a designed low-power system, as shown in Table 2. Therefore, the system turns on the current flow only for the short time needed to measure the voltage across the bridge. Moreover, the user can reduce the current by selecting a strain gauge with higher nominal resistance and reducing the voltage, but in some cases, it could imply a reduction in accuracy. A MOSFET transistor, controlled by the GPIO line of the microcontroller, is used to switch the current flow. The change in power supply source controlled by a transistor and reference voltage source is carried out by means of jumpers. The voltage across the EX+ and EX− terminal is used as a reference voltage for the ADC. The system can operate with quarter- (shown in Figure 11), half-, and full-bridge configurations (shown in Figure 12), but the first two setups need the installation of additional resistors to complete the bridge.

**Figure 13:**
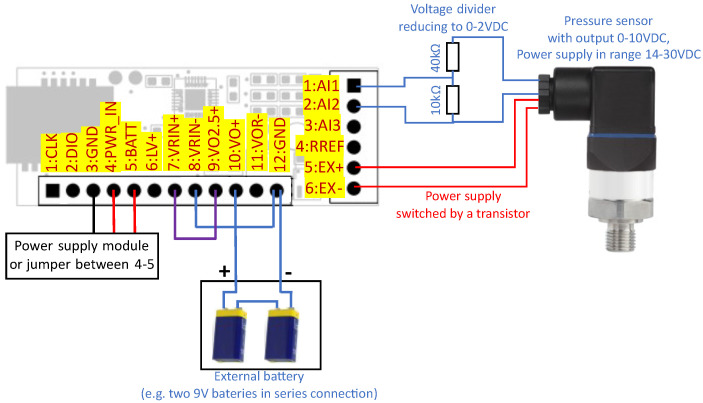
Operation with voltage output pressure sensor and external batteries (18 V in total).

The same transistor can control a power supply with a voltage of up to 30 V, which makes it possible to operate with integrated sensors, e.g., a pressure sensor with a 14–30 V power supply and voltage output, as shown in Figure 13. In that configuration, the system uses two independent batteries for the sensor and transducer. The integrated sensor is turned on for the time needed to perform the measurement. To operate with voltage outputs above 2.5 V (the maximum value for ADC), a voltage divider has to be implemented. It is also possible to operate with current outputs using voltage drop measurement on the additional resistor, but that setup could consume more energy in comparison to the sensor with voltage output. The tested voltage output pressure sensor needs several mA during constant operation. For the current output sensor (4–20 mA), it could be more than 20 mA.

The measurement methods presented above can be used not only to support the mentioned basic types of sensors used in aviation laboratories. There are many sensors based on these types of measurements, e.g., resistance measurement is used in potentiometric position sensors, strain gauges are used in some pressure and vibration sensors, and voltage measurement can support most voltage output sensors. Providing the possibility of connecting an external battery and controlling the sensor power supply according to the program also increases the capabilities of the system to operate with many types of sensors.

## 3. Test Results

The basic result of the research is a designed transducer examined in a series of tests, as described in the following sections. Outcomes from tests of operation with sensors, wireless power transfer, battery power supply, and network communication show the final capabilities of the designed smart transducer. The presented configurations are the implementation of measurement methods presented in the previous chapter.

### 3.1. Test of Operation with Sensors

**Figure 14:**
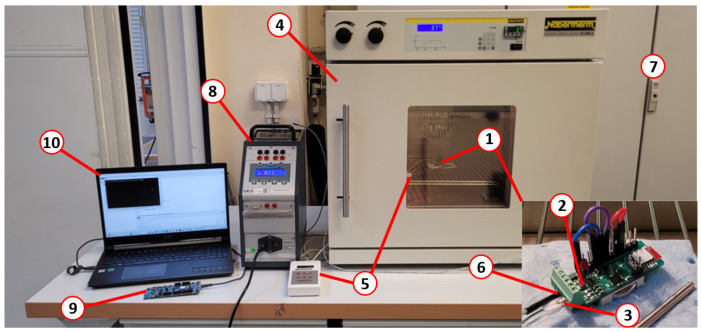
Test of operation with thermocouple.

**Figure 15:**
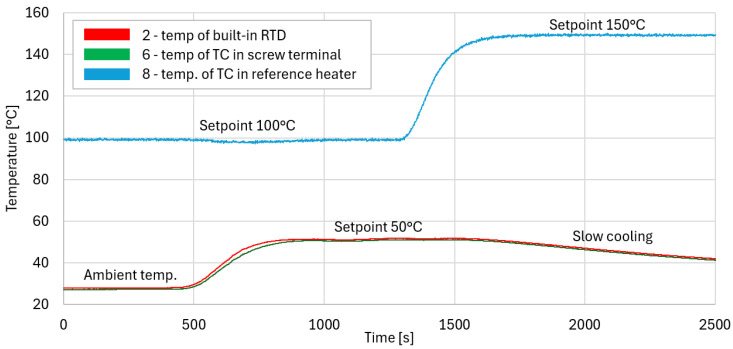
Results from test of operation with thermocouple.

The correct operation of thermocouples and RTDs was confirmed in the series of experiments. First, to prove the operability of the temperature compensation transducer (1) with a built-in RTD (2) and thermocouple (3) connected to the input, they were installed in a heater (4), as shown in Figure 14. The temperature inside the heater was measured by a reference temperature meter (5) and thermocouple (6) installed in the screw terminal of the transducer and connected to the laboratory data acquisition system (7). The measuring junction of the thermocouple connected to the transducer was installed in a reference heater (8). That configuration made it possible to change temperatures on both thermocouple ends and simulate the real operation of the system. Data were registered wirelessly through the nRF52840 development module (9) on the computer (10). The transducer was tested in the range of 25–60 °C with a thermocouple in the range of 25–300 °C. In one of the test cases, the temperature set point for the heater was increased from ambient temperature to 50 °C when the reference heater was set to 100 °C. Next, the set point for the reference heater was increased to 150 °C during transducer cooling, as demonstrated in Figure 15. Constant readings of the temperature at both set points (100 °C and 150 °C) prove the correct operation of the system. The test also proved the correct operation of the RTD sensor used for compensation. Similar tests were performed for the RTD sensor connected to the screw terminal. For both tested types of sensors, accuracy was better than 1 °C, and it could be easily improved by adjustment for specific sensors.

**Figure 16:**
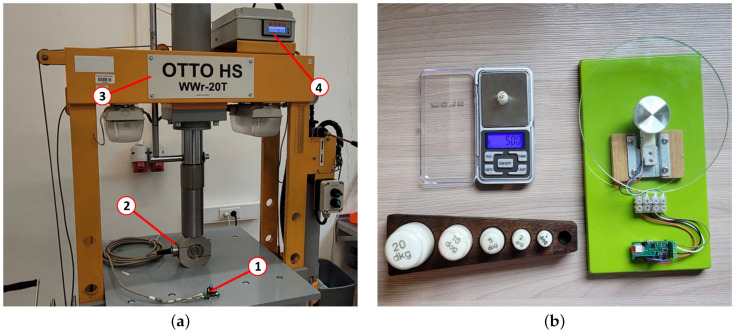
Tests with strain gauges: (**a**) load cell on a press; (**b**) strain gauge-based scale.

The operation of the transducer with strain gauges was also proven in two tests. The first transducer (1) was connected to a strain gauge-based load cell (2) pressed by a press (3) with a reference gauge (4), as shown in Figure 16a. In the second test, the operation of the transducer with a strain gauge-based scale was examined by comparison with a high-accuracy reference scale, as demonstrated in Figure 16b. In both cases, the excitation voltage was used as a reference voltage.

**Figure 17:**
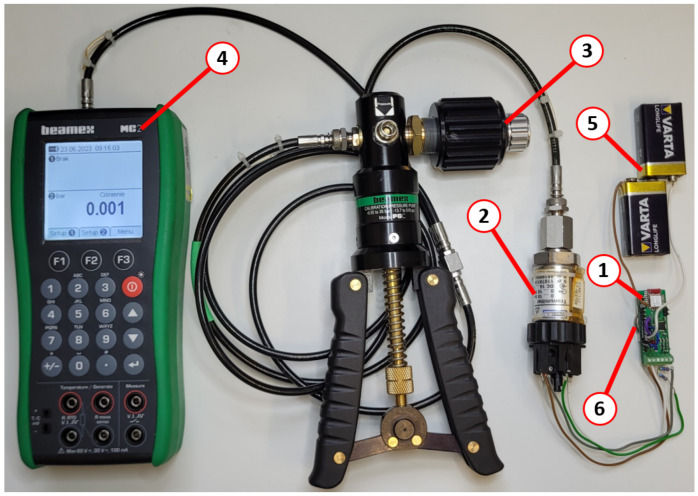
Test of operation with voltage output sensor.

The last tested group consists of sensors with voltage output. To examine these features of the transducer (1), the pressure sensor (2) with voltage output was connected to a pressure calibration system with a precise pump (3) and reference gauge (4), as shown in Figure 17. The system was powered by two 9 V batteries (5) connected in series to reach 18 V of power supply for the sensor and one CR2032 battery (6) to supply the transducer. The sensor output was reduced from 0–10 V to 0–2 V by the voltage divider. The series of tests proves the operability and high system accuracy of the transducer.

### 3.2. Test of Battery Power Supply

**Figure 18:**
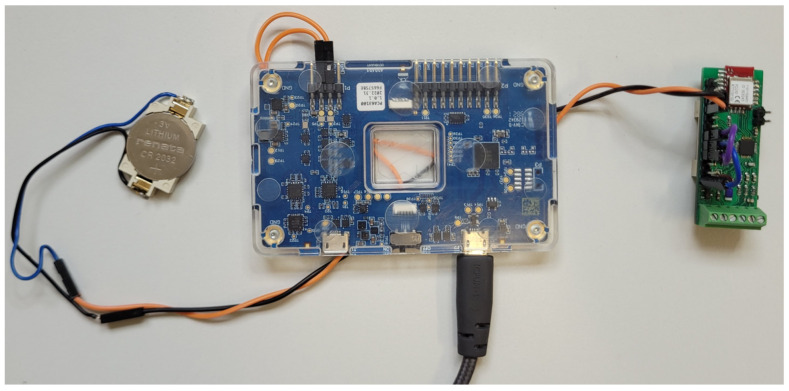
nRF Power Profiler Kit II used during power optimization of the transducer.

**Figure 19:**
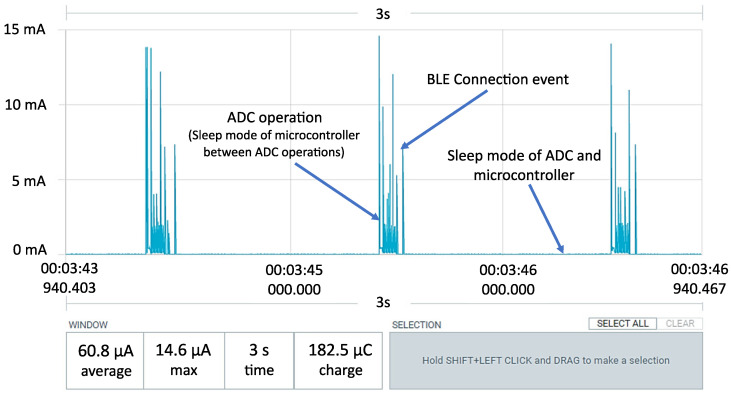
Result of power optimization for operation with PT1000 sensor (1 Hz).

Power consumption is strongly dependent on the settings of communication and measurements. The main rule is that higher frequencies cause higher power consumption. Figure 18 shows the test setup with the Nordic Power Profiler Kit, which was used to easily compare the effects of different settings. This single-board system can work as a power supply with high-precision current measurement or as an ampere meter. The measurements are visible on a dedicated computer application, as shown in Figure 19. In the test configuration, the power profiler measures the current flowing between the battery and the base module. The tested configurations of the system were the same as those presented in Section 2.5 (Figure 8, Figure 9, Figure 12 and Figure 13) and Section 3.1 (Figure 14, Figure 16b and Figure 17). The tests were performed in the basic configuration of the transducer without the WPT module.

The power requirements of the transducer were examined for measurement frequencies in the range of 0.1–2 Hz and various power supply configurations, as presented in previous sections. The system reduces power consumption by switching the ADC and microcontroller to sleep mode between operations. The presented results are the outcomes of energy consumption optimization to date, but the research will be continued. The results of power optimization and estimated battery operation time for the thermocouple, RTD, and strain gauge are presented in Table 3. Calculations assume 85% of operation time resulting from the capacity of CR2032 because the voltage of that coin cell goes below 2.7 V (limit of the designed transducer) before full discharge. Calculations do not take into account the self-discharge effect of batteries and show only the approximate operation time of the transducer based on battery capacity.

In both tested ways of measuring temperature, the system generates an excitation current, which is set to 50 μA. The power consumption is higher for the thermocouple because the system has to measure the voltage on the thermocouple and the temperature of the in-built RTD for compensation, which causes longer operation out of sleep mode. Reducing the frequency of compensation temperature measurements could increase the power efficiency, but it would also reduce the measurement accuracy when the system works in a variable-temperature environment. A transistor switched the excitation voltage of the strain gauge, which enabled the system to reach reasonable power consumption.

**Figure 20:**
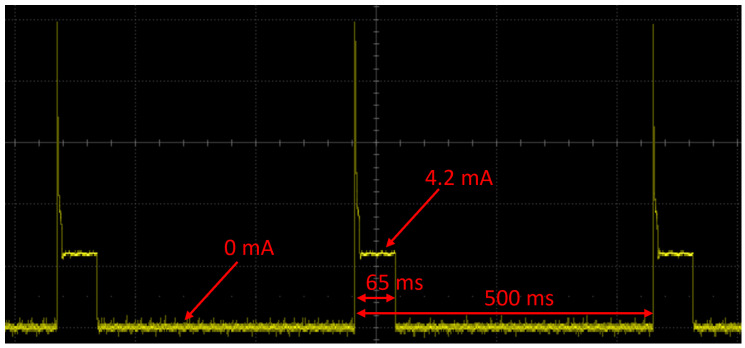
Current consumption for a pressure sensor switched with a frequency of 2 Hz.

The performed research also shows operation possibilities with voltage output integrated sensors and two independent power sources for the transducer and sensor. As introduced in the previous chapter, the in-built transistor switched the power supply. The current consumption of the sensor is higher immediately after turning on the power, as shown in Figure 20. This is probably the effect of capacitor charging and voltage stabilization operations. Current consumption in constant operation is equal to 4.2 mA. In the proposed approach, the sensor is switched on only for 65 ms, so the power consumption changes with the frequency of switching, as shown in Table 4. The Power Profiler Kit was used to measure the current of the transducer, but it cannot be used for voltages above 5 V. For this reason, the voltage measurement on an additional resistor installed in series was used to measure the pressure sensor current. The voltage waveform was recorded using an oscilloscope and converted into a current value according to Ohm’s law. The average current during 65 ms of operation is equal to 4.8 mA.

### 3.3. Test of Wireless Power Transfer

**Figure 21:**
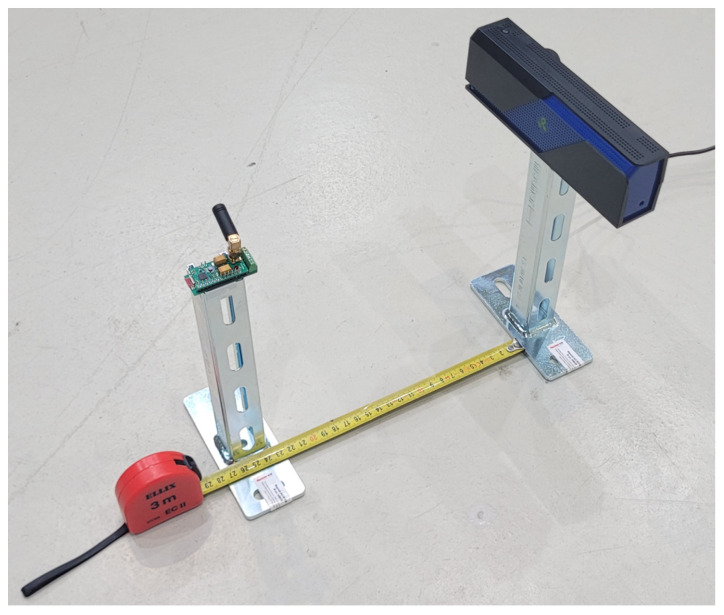
Test setup prepared for WPT range test.

To find the limit of the WPT range, the system was also tested with the designed extension board. Figure 21 shows the setup prepared for the WPT test. First, the system was tested without a battery. The distance between the transducer with the WPT module and the transmitter increased slowly until the system lost the BLE connection. The test was performed for various measurement frequencies of the system during operation with an RTD, thermocouple, and strain gauge-based scale (full bridge configuration), as shown in Table 5. The WPT range decreases with the increase in frequency because there is not enough time to charge capacitors at higher frequencies. The relation between the power consumption of the transducer (shown in Table 3) and the WPT operation range is not easy to interpret. Power consumption with the WPT module is higher than operation only with a battery (without the module) because of voltage conversion and capacitor current leakages. Moreover, conversion efficiency changes with voltage and current. Average current is a significant factor in power consumption, but the time of operation out of sleep mode and maximum current peak value are also important. For example, a short, steep increase in current could discharge capacitors even if the average current is low. Next, tests were performed with the installed battery. Outside of the full WPT operation zone, the transducer uses the RF source and battery. In each cycle, it switches from capacitors to batteries when capacitors are discharged. WPT usage decreases with increasing distance until the system starts using only batteries.

### 3.4. Test of Operation in a Real Test Environment

**Figure 22:**
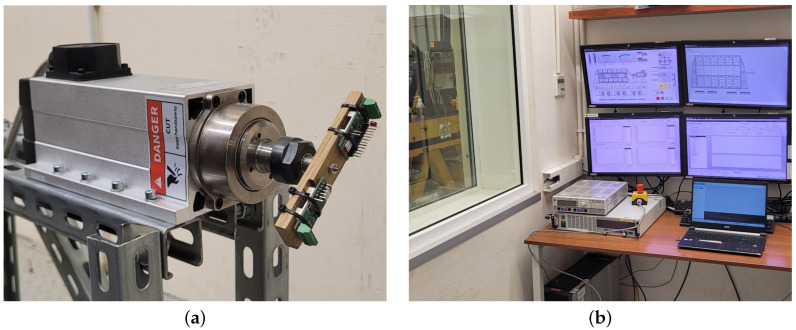
Test configuration: (**a**) transducers installed on the rotary shaft; (**b**) control room.

**Figure 23:**
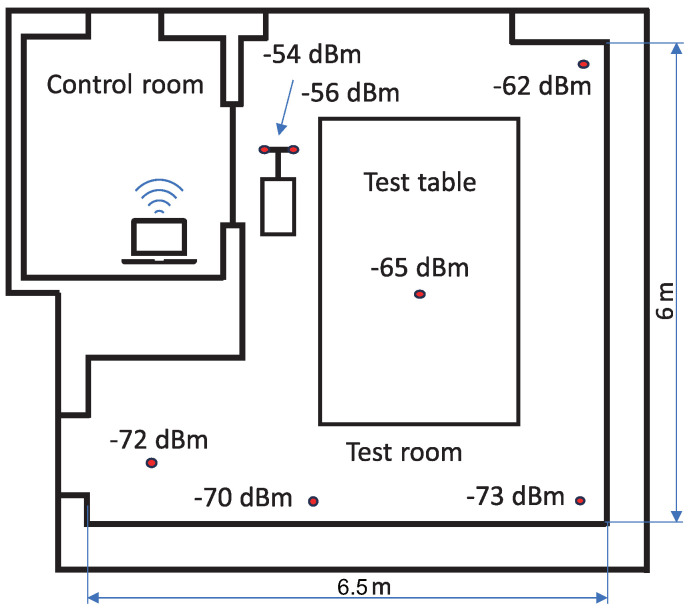
RSSI factors corresponding to the transducers in the test room.

In the final phase of the research, the designed transducer was tested in a laboratory environment in the Component Test Laboratory. Two modules were installed on the rotary element to prove wireless communication under these conditions, as shown in Figure 22a. The test was performed up to 5000 rpm with a balanced rotor. This situation may reflect the conditions during the tests of the propeller drive chain. Transducers wirelessly transmit the temperature measured by the internal PT1000 temperature sensors. The next five transducers were placed on the installation in various locations in the test room. All BLE devices were connected to the computer with the BLE module placed on the desk inside the control room, as shown in Figure 22b, where the operator worked during the test. Data were available through the “nRF Connect for desktop” application. Measurements were carried out at a frequency of 1 Hz. First, the system was tested only with a battery power supply; next, the designed extension module was connected to prove the WPT operation of rotating modules. The test shows that the system was operable during rotation without any visible changes in the accuracy of measurements and the wide range of BLE in the test room. Communication between BLE devices and the data gate was possible through a wall with double bulletproof glass. Rotating modules could be powered wirelessly from a distance of 0.35 m. That range was lower than the result of the static test for the same frequency because the transmitted power is dependent on the angle between the antennas. The research also included checking the RSSI (Received Signal Strength Indication coefficient) for individual BLE module locations, as shown in Figure 23.

RSSI values between −54 dBi and −73 dBi show the good strength of the signal. The test results prove the operability of the system in a star network consisting of seven transducers, but this is not the limit of the system. According to the nRF52840 data sheet and selected soft device (S140) documentation [29,35], it should be possible to build a star network consisting of twenty devices, which will be investigated in future research.

## 4. Discussion

The main goal of constructing a multipurpose, wireless, low-power transducer has been achieved. The test results showed that the designed transducer can support all planned types of sensors with high measurement accuracy. What distinguishes the proposed system is its very low energy consumption, enabling operation for many months on battery power, and wireless radio power supply. Currently available telemetry systems offer short battery life or wireless inductive power from a distance of a few millimeters. The range of the wireless power supply in the tested configuration is not far, but it is enough to power transducers working with low frequencies on the test table or rotating from a distance of several dozen centimeters. This approach could be interesting when the test has to be prepared quickly or research includes long-term testing of moving or rotating components. Tests have shown that rotation of the transducer does not make BLE communication and wireless power transfer through the radio impossible. In both stationary and rotary applications, the combination of wireless and battery power provides much more reliability than a purely wireless power supply. The user does not have to worry about unintentionally blocking access to the energy transmitter or going out of the wireless power range. Research also shows a wide range of BLE communication possibilities. Transducers could communicate not only in the area of the test room but also through the walls. In the most straightforward test campaigns, the computer in the control room with BLE support may play the role of a central point for a star network and could be used for visualization or data analysis, as was introduced in the last test.

It should be noted that this is a prototype without a housing and has not yet been tested in terms of resistance to environmental conditions, except for the presented temperature compensation test. Environmental tests will be completed after the housing has been designed; however, the designed transducer is not dedicated to tests performed during aircraft flight and will not be tested under extreme temperature and pressure conditions. According to the data sheets of its components, the working temperature of the transducer is limited to 85 °C (limit for Proteus III communication module).

The transducer was designed for low sampling frequencies; therefore, it is not appropriate for all types of measurements, e.g., if we wanted to perform measurements in each rotation of the shaft. Measurements are performed at time intervals corresponding to communication via BLE and are not synchronized between the transducers. The proposed approach is suitable for measuring parameters that change at low frequencies.

Future research will be focused on the network capabilities of the system and determining the possibilities for a low-power design approach to battery power supply of the actuators used in aircraft component testing laboratories, e.g., the valves used for oil and airflow control. In parallel, research is also being carried out on multi-channel wireless transducers dedicated to testing high-speed rotating components, e.g., jet engine bearings.

## 5. Conclusions

This paper presents a new approach to aircraft components test rig instrumentation based on the use of a wireless transducer and a combination of battery and wireless power supply, with a low-power design approach. The operability and measurement accuracy were proven during a series of tests in various sensors and power source configurations. Test results show that the designed smart transducer meets all defined requirements in terms of measurement accuracy, long battery operation time, and radio wireless power transfer, which distinguishes it from other available telemetry systems. The proposed approach will speed up the preparation of test instrumentation and simplify test infrastructure reconfigurations because of the lack of wires. Moreover, test heads and test rigs could be easily moved between laboratories with installed instrumentation and wireless transducers without the need for the preparation of sensor wiring.

Measurements carried out in aviation laboratories directly impact the operational safety and improvement of aircraft components. Therefore, searching for new and more efficient research methods that allow for exploration beyond the limits of basic procedures that have been used for years is of great importance. This paper fills one of the gaps in the research on wireless, low-power, smart transducers in crucial areas of the industry.

## Figures and Tables

**Table 1:** Transducer pinout.

Pin Terminal		Screw Terminal	
1: CLK	Clock pin used for programming	1: AI1	Analog input 1
2: DIO	Input/output for programmer	2: AI2	Analog input 2
3: GND	GND	3: AI3	Analog input 3
4: PWRIN	Power input	4: RREF	Input to reference resistor
5: BATT	Voltage from battery	5: EX+	Excitation voltage+
6: LV+	Not used	6: EX–	Excitation voltage-
7: VRIN+	MAX11410 reference coltage input+		
8: VRIN–	MAX11410 reference voltage input-		
9: VO2.5+	Reference voltage output		
10: VO+	Input of voltage switched by transistor		
11: VOR–	Voltage before transistor for reference		
12: GND	GND		

**Table 2:** Current consumption for constant operation with different sets of resistance and voltage.

Strain Gauge Nominal Resistance	1.8 VDC	3.3 VDC	10 VDC
120 Ω	15 mA	27.5 mA	83.3 mA
350 Ω	5.1 mA	9.4 mA	28.5 mA
1000 Ω	1.8 mA	3.3 mA	10 mA

**Table 3:** Current and estimated operation time for sensors operating with CR2032 battery.

	PT1000		Thermocouple		SG Bridge (1000 Ω, 3 V)	
Frequency	Average Current	Time	Average Current	Time	Average Current	Time
Hz	μA	Days (24 h)	μA	Days (24 h)	μA	Days (24 h)
0.1	18	453	23	354	38	214
0.2	22	370	36	226	62	131
0.5	38	214	66	123	163	50
1	60	136	118	69	197	41
2	114	71	236	35	480	17

**Table 4:** Current and operation time for pressure sensor supplied by two 6LR61 batteries.

	Transducer Power Supply CR2032 230 mAh		Sensor Power Supply-Two 6LR61 9 V Batteries, 550 mAh	
Frequency	Average Current	Time	Average Current	Time
Hz	μA	Days (24 h)	μA	Days (24 h)
0.1	15	543	31	734
0.2	19	429	62	367
0.5	30	271	156	146
1	47	173	312	73
2	92	89	624	36

**Table 5:** Results of WPT operation range test.

	WPT Distance [cm]		
Frequency [Hz]	RTD PT1000	Thermocouple	Strain Gauge Bridge
0.1	115	64	62
0.2	102	59	57
0.5	83	54	50
1	73	48	42
2	62	43	30

## Data Availability

Data are contained within the article.
